# Child and Parent Perspectives on Daily Functioning after Perinatal Brain Injury

**DOI:** 10.1016/j.jpedcp.2025.200172

**Published:** 2025-08-07

**Authors:** Lisanne M. Baak, Corline E.J. Parmentier, Nienke Wagenaar, Fleur-Sophie N. Buijsen, Anne A.M. van Wendel de Joode, Jan Willem Gorter, Manon J.N.L. Benders, Floris Groenendaal, Linda S. de Vries, Cora H. Nijboer, Thomas Alderliesten, Corine Koopman-Esseboom, Cornelia H. Verhage, Rian M.J.C. Eijsermans, Niek E. van der Aa, Marjolijn Ketelaar

**Affiliations:** 1 Department of Neonatology, University Medical Center Utrecht Brain Center and Wilhelmina Children's Hospital, Utrecht University, Utrecht, The Netherlands; 2 Neuropsychology Section, Department of Pediatric Psychology, Wilhelmina Children's Hospital, University Medical Center Utrecht, Utrecht University, Utrecht, The Netherlands; 3 Department of Rehabilitation, Physical Therapy Science & Sports, UMC Utrecht Brain Center, University Medical Center Utrecht, Utrecht, The Netherlands; 4 De Hoogstraat Rehabilitation, Center of Excellence for Rehabilitation Medicine, UMC Utrecht Brain Center, University Medical Center Utrecht, Utrecht, The Netherlands; 5 Department for Developmental Origins of Disease, University Medical Center Utrecht Brain Center and Wilhelmina Children's Hospital, Utrecht University, Utrecht, The Netherlands; 6 Child Development and Exercise Center, University Medical Center Utrecht, Utrecht University, Utrecht, The Netherlands

**Keywords:** neonatology, developmental health, perinatal brain injury, psychosocial health, daily functioning, questionnaire study

## Abstract

**Objective:**

Perinatal brain injury can result in long-term neurodevelopmental sequelae. To examine the most significant consequences from a patient perspective, this questionnaire study explored the strengths and difficulties in daily functioning after perinatal brain injury, by child self-report and parent-proxy report.

**Study design:**

Cross-sectional questionnaire study of participants ≥8 years of age born with hypoxic-ischemic encephalopathy treated with therapeutic hypothermia, or with perinatal arterial ischemic stroke, and their parents. Open-ended questions regarding strengths and difficulties in daily functioning were analyzed by thematic analysis using the International Classification of Functioning, Disability and Health as coding framework.

**Results:**

Of the 102 participants (hypoxic-ischemic encephalopathy: n = 50, perinatal arterial ischemic stroke: n = 52) with a median age of 12.4 (range 8-25) years, 81% of the questionnaires (n = 83) were filled out by both participants and parents. Participants mainly reported participation in recreational activities and school-related learning abilities as strengths, while parents most frequently highlighted their child's personality traits. Difficulties were reported by 91% of participants and 76% of parents. Both parents and participants often mentioned difficulties with cognitive functioning including attention, memory, and processing speed. Furthermore, parents described social skills and movement difficulties, while participants mostly reported learning difficulties. Half of the parents reported that the difficulties significantly impacted their families' daily lives.

**Conclusions:**

Children and young adults with a history of perinatal brain injury face a variety of challenges in their everyday lives, emphasizing the importance of taking the full spectrum of sequelae into account in care, counseling, and future research.

Perinatal brain injury is a major cause of long-term neurodevelopmental problems. The primary causes among (near-)term infants include hypoxic-ischemic encephalopathy (HIE) due to presumed perinatal asphyxia and perinatal arterial ischemic stroke (PAIS).^1^

Long-term neurodevelopmental consequences following perinatal brain injury often extend beyond motor impairments and may involve cognition, behavior, sensory processing, and epilepsy.^2^, ^3^, ^4^, ^5^ While the patients' perspectives are increasingly valued in health care research, data on the consequences of perinatal brain injury from the perspectives of the children and their families remain unstudied. However, these perspectives can reveal challenges that are under-recognized in research or routine care, as patients and families may prioritize different aspects of functioning than professionals. This was demonstrated in a study of children with cerebral palsy (CP), where professionals, children, and parents each emphasized different aspects of functioning underscoring the need to integrate multiple viewpoints.^6^

Understanding the possible consequences of perinatal brain injury from the perspectives of the children and their families is crucial to tailor follow-up care, improve parental counseling and education, set research priorities, and determine relevant outcome measures. Therefore, we aimed to describe the self-reported and parent-proxy–reported daily functioning of children aged ≥8 years with a history of PAIS or HIE treated with therapeutic hypothermia (TH).

## Methods

We performed a cross-sectional online questionnaire study on children, adolescents, and young adults ≥8 years of age with a history of perinatal brain injury (further referred to as “participants”), along with their parents and/or caregivers. Answers to the open-ended questions were analyzed using the framework of the pediatric version of the International Classification of Functioning, Disability and Health (ICF-CY) to systematically explore functional categories that hold significance for participants and their parents in their daily functioning.^7^ This study followed the Standards for Reporting Qualitative Research.

### Participants

Participants were selected from the Neonatal Stroke Registry Utrecht (NSRU) and the Data Registry of infants with Perinatal Asphyxia (DRPA). They were either admitted to the Neonatal Intensive Care Unit or attended the follow-up clinic of the Wilhelmina Children's Hospital, the Netherlands, from 1998 onward. For the NSRU, parents provided written informed consent for their child's participation in the registry (reference number 21/660). For the DRPA, written informed consent was obtained from parents of children who were still in follow-up. The Ethical Review Committee waived the requirement for informed consent for those discharged from follow-up (reference number 22/898). For this questionnaire study (ref. 212209297), parents gave informed consent for participants under the age of 16, with participants aged ≥12 until 16 years providing additional informed consent.

Eligible participants had either HIE treated with TH or PAIS, according to previously described definitions,^8^^,^^9^ were ≥8 years to allow self-report, and able to understand English or Dutch. To keep the sample representative of the population, there were no exclusion criteria. Neonatal, neuroimaging, and neurodevelopmental follow-up data were collected from the NSRU and DRPA as previously described.^5^^,^^10^ Information on the neurodevelopmental tests used at the outpatient clinic is provided in Appendix 1. A developmental delay was defined as a standardized test score ≤-1 SD. Socioeconomic status was determined using SES-WOA scores from Statistics Netherlands.^11^

### Questionnaire

The online questionnaire, designed by researchers in the field of perinatal brain injury (L.M.B., N.E.A.) and pediatric rehabilitation (M.K.), explored strengths, difficulties, and concerns experienced in daily life. It included 3 open-ended questions for children, and 4 for parents and participants ≥16 years written at an intermediate (B1) language level according to the Common European Framework of Reference for Languages (Appendix 2). The questions were intentionally crafted to capture a wide spectrum of important themes for both participants and parents, avoiding any bias towards particular domains or topics. Respondents were not given example responses and were encouraged to report as many or as few topics as they felt relevant, with no minimum answer length required. Several parents of children with perinatal brain injury provided feedback in the creation process of the questionnaire. Invitations with a personal access link were sent via email to parents or to participants when aged ≥16 years, and online consent was obtained. Parents completed their questionnaire first, followed by the child. Children were instructed that their parents could help if needed but were asked to report their own experience. Participants aged ≥16 years could choose whether their parents also completed a questionnaire.

### Data Analysis

Answers were coded using the ICF-CY, developed by the World Health Organization.^7^ The ICF-CY is a comprehensive internationally-recognized framework that systematically describes health components of developing children and youth and the influence of environmental factors. It is particularly suitable for capturing the complexity and multidimensionality of functioning following perinatal brain injury and aligns with the perspectives of patients and family members. The alphanumeric code structure describes letter-coded domains of functioning: body functions (b), body structures (s), activities and participation (d), and personal and environmental factors (e), followed by a hierarchical numeric code providing up to 4-digit specification. Responses were coded to 101 different codes up to at least the third-level digit, using established linking rules.^12^ As some personal factors are not classified by the ICF-CY due to the large social and cultural variance associated with them, we created 5 additional codes (f) for categories absent in the ICF-CY (sensory processing; independence; confidence; humor; empathy, overall physical health). Additionally, we exploratively analyzed the impact of the reported difficulties on daily life (Q3) and whether there was a concern or barrier for development (Q4). Since ICF-CY was less suitable to analyze these perspectives, we applied a 2-step approach combining binary coding (presence/absence of impact or concern) with qualitative, inductive open coding. To ensure data validity, reliability, and generalizability, 2 reviewers (L.M.B., C.E.J.P.) coded answers independently, reaching consensus upon discussion and if necessary, by consulting a third reviewer (M.K.).

### Statistical Analysis

Clinical characteristics were presented as numbers and percentages or as medians with IQR. ICF-CY category distribution was presented descriptively up to the fourth-digit level and as percentages of the cohort. Data were analyzed with SPSS Statistics, version 29.0.1 (IBM).

## Results

### Participants

Of the 104 eligible participants with HIE treated with TH and 152 with PAIS, 50 (48%) and 52 (34%) children, adolescents, and young adults and/or their parents participated in this study, respectively (Table I). When comparing participants with eligible nonparticipants, no significant differences in neonatal and magnetic resonance imaging (MRI) characteristics were observed except for a lower gestational age among the nonparticipants compared to the participants with PAIS (median [IQR] 38 [7^+2^] vs 39^+6^ [3^+4^] weeks, *P* = .004). Of the participants, 5 (10%) with HIE and 8 (15%) with PAIS were born preterm (median gestational age 35^+6^ [IQR 1^+4^] and 31 [IQR 3^+4^] weeks, respectively). Eleven (21%) PAIS participants had perinatal asphyxia without HIE and TH, and 2 participants treated with TH for HIE had concomitant PAIS. In one of these children, PAIS was the predominant injury pattern on neonatal MRI.

**Table I:** Clinical and neurodevelopmental outcome characteristics of the study cohort

	PAIS (n = 52)	HIE (n = 50)
Neonatal characteristics		
Female	31 (60%)	20 (40%)
Gestational age, weeks	39^+6^ (3^+4^)	40^+2^ (2^+5^)
Birth weight, g	3300 (890)	3500 (795)
Birth weight *z* score	−.10 (1.39)	.01 (1.54)
Apgar score at 5 min	9 (2)	4 (2)
Socioeconomic status (SES-WOA)	0.11 (0.28)∗	0.16 (0.23)
Perinatal asphyxia	11 (21%)	50 (100%)
Modified Sarnat stage		
Mild	9 (17%)	15 (30%)
Moderate	2 (4%)	34 (68%)
Severe	0 (0%)	1 (2%)
Comorbidities†	12 (23%)	20 (40%)
Neonatal MRI characteristics		
Predominant pattern of injury		
Normal	0 (0%)	18 (36%)
Mild WMWS	0 (0%)	9 (18%)
Moderate-severe WMWS	0 (0%)	7 (14%)
Mild DGM	0 (0%)	1 (2%)
Moderate-severe DGM	0 (0%)	2 (4%)
Near total injury	0 (0%)	1 (2%)
Subdural hematoma or intraventricular hemorrhage	0 (0%)	11 (22%)
Stroke	52 (100%)	1 (2%)
Anterior branch MCA	1 (2%)	0 (0%)
Main MCA	8 (15%)	0 (0%)
Middle branch MCA	4 (8%)	0 (0%)
Posterior branch MCA	8 (15%)	0 (0%)
Cortical stroke	4 (8%)	0 (0%)
Perforator stroke	17 (32%)	1 (2%)
PCA/ACA	9 (17%)	0 (0%)
Cerebellar stroke	1 (2%)	0 (0%)
Neurodevelopmental follow-up		
Age at last follow-up visit, years	5.6 (0.6)	8.4 (3.9)
CP	12 (23%)	3 (6%)
GMFCS level I-II	12 (23%)	1 (2%)
GMFCS level III-V	0 (0%)	2 (4%)
Motor developmental delay,	2 (4%)	10 (20%)
Cognitive delay	6 (12%)∗	12 (24%)∗
Language delay	7 (14%)	5 (10%)
Epilepsy	4 (8%)	2 (4%)
Behavioral problems	10 (19%)‡	15 (30%)
Audiovisual deficit related to perinatal brain injury	8 (15%)	4 (8%)
No diagnosed delays or problems,	27 (52%)	24 (48%)
Special education§	8 (15%)	9 (18%)

*ACA*, anterior cerebral artery; *DGM*, deep gray matter; *GMFCS*, gross motor function classification system; *MCA*, middle cerebral artery; *PCA*, posterior cerebral artery; *WMWS*, white matter watershed injury.

Values are depicted as either n (%) or as median (IQR).

∗ n = 1 missing.

† Comorbidities included: Down syndrome (n = 2), Turner syndrome (n = 1), Scimitar syndrome n = 2, cystic fibrosis (n = 1), long QT syndrome (n = 1), brain tumor (n = 1), neonatal meningitis (n = 4), sepsis with positive blood culture (n = 3), persistent pulmonary hypertension (n = 12), necrotizing enterocolitis (n = 1), twin-to-twin transfusion syndrome (n = 2), fetal intracranial hemorrhage (n = 1), tension pneumothorax (n = 1), congenital cataract (n = 1), severe fetomaternal transfusion with partial exchange transfusion (n = 1), and/or hydronephrosis requiring surgery shortly after birth (n = 1).

‡ n = 2 missing.

§ n = 4 with PAIS and n = 2 with HIE that were last seen before attending primary school.

The socioeconomic status of the families as estimated by the SES-WOA scores was slightly above average. At the last clinical follow-up visit (median age 5.9 [range 1.5-12.8] years), approximately half of the participants did not show any delay on the tested outcome domains.

The median age when filling out the questionnaire was 12.4 years (PAIS median 13.9 [range 8.0-25.4], HIE median 11.2 [range 8.6–15.3] years). Eighty-three (81%) questionnaires were filled out by both the participant (self-report) and the parent (parent-proxy report), 11 (11%) were parent-proxy reports only, and 8 (8%) self-reports only (all adults with PAIS). The reporting frequency of strengths and difficulties were summarized in 1-level digit ICF-domains (Appendix 2) and 4-level digit ICF-categories (Table II).

**Table II:** Top-mentioned strengths and difficulties in daily functioning coded to ICF-CY categories and sorted by reporting frequency of the participants and their parents

### Strengths

Findings were mostly mapped to the ICF domains body functions (mental functions) and activities and participation (learning and applying knowledge; community, social, and civic life; and interpersonal relationships and interactions, Appendix 2). Parents reported strengths in median (IQR) 3 (2) different domains, children in 2 (2), respectively. Analyzing the ICF-coded categories up to fourth-digit level, participants often emphasized their participation in recreational activities and school-related learning activities. Parents frequently highlighted their child's personality, social skills, and learning abilities (Table II). Five participants and 2 parents were unable to describe any strengths. Participant/parent quotes are provided in Table III.

**Table III:** Examples of quotes from the participants and their parents on their strengths, difficulties, and the impact on their daily lives

Strengths
Parent, 12-15 years with HIE: *Sociable, sweet, learns easily from the principle “monkey see, monkey do”. Laughs a lot and is creative.*
Participant, 18+ with PAIS: *My strengths are my perseverance, willpower and humor. I try to look at things in a positive way and think of solutions for the things that I find hard.*
Participant, 8-12 years with PAIS: *Handball, making friends, playing*
Parent, 16-18 years with PAIS: *He has grit, is very creative, works hard and is very attentive.*
Difficulties
Parent, 8-12 years with HIE: *He easily forgets things. He does not have a good memory, at least short term. He has been taking 4 years (including a COVID-19 break) to get his swimming certificate. The teacher says his legs are not strong enough and he is not able to perform certain movements. He still has pee accidents during the day. He does not feel it. Additionally, very often I have to specifically name and explain things to him that are obvious for other children. He wants to be liked and is therefore easy to manipulate and abuse.*
Parent, 8-12 years with HIE: *When something does not suit him he is sometimes extremely explosive; he has a short fuse. He has difficulty concentrating, and avoids problems when they get difficult. He struggles with automating skills.*
Participant, 12-15 years with HIE: *I find memorizing things and calculating very hard. I find it hard to understand other people, sometimes they look angry when they are thinking and then I think they are angry at me. I don't like when I have to switch quickly, then I don't see the overview anymore and I get angry or afraid. I would really like to go to ballet, but it's so fast, I can't do it and I think that's a real shame. I used to play korfball, but I quit because the game has to be so fast and then the team lost because of me.*
Participant, 12-15 years with HIE: *I forget a lot and always feel the urge to say everything that is on my mind out loud.*
Parent, 8-12 years with PAIS: *She has limited fine motor skills in her right hand. And she has a learning disability.*
Parent, 16-18 years with PAIS: *Very fine motor skills of the left hand, such as buttoning, or carefully tying laces. The average level of concentration and planning tasks is low. He does not find it easy to make and sustain friendships with peers. He seems somewhat younger in his behavior.*
Parent, 16-18 years with PAIS: *He has a low energy level and is easily overstimulated. He quickly reaches his mental limit. He cannot see on the right (hemianopsia), controlling his right half of the body takes more effort and there is also more body tension on the right.*
Participant, 18+ with PAIS: *Nothing that is currently bothering me… I am only twenty years old now, so I cannot know what the future will hold, but for now I am in perfect health.*
Impact on daily life
Parent, 12-15 years with HIE: *She attends special education, which is quite significant. She takes a taxi to school and doesn't have any friends nearby. Her ability to be independent outside the home is limited, and although she participates in the G-team for athletics and volleyball, she is losing connection with her peers. At home, she needs help with many tasks. She can do a lot on her own, but without direction, she struggles and doesn't accomplish much.*
Parent, 8-12 years with HIE: *Both at school, in interactions with others, and at home, he encounters certain challenges. He seeks a lot of attention and reassurance from us. Sometimes he needs explanations for things that happen in life. He also requires extra guidance in interacting with others, along with more structure and clarity.*
Parent, 8-12 years with PAIS: *She mainly has outbursts of anger at home, which significantly impacts our family, especially with her 3 sisters. She becomes overstimulated quickly and reacts strongly to other family members.*
Parent, 12-15–year-old with PAIS: *She clearly performs below her intelligence level. At home, she is energetic, but she becomes shy in unfamiliar situations. She struggles with planning and winding down in the evening. Her life is quite busy, and keeping appointments is problematic; she often forgets them altogether.*
Participant, 18+ with PAIS: *It makes me quite tired and irritated. People around me notice it. I was also bullied for it in elementary school.*
Participant, 18+ with PAIS: *People think I don't speak the language, that I'm not interested, or that I'm not intelligent enough to keep up with them.*

### Difficulties

The main ICF domains in which difficulties were reported were body functions (mental; neuromusculoskeletal and movement-related functions), and activities and participation (learning and applying knowledge; community, social and civic life; interpersonal relationships and interactions, Appendix 2). Parents reported difficulties in median (IQR) 2 (2) different domains, children in 1 (1), resp. Both parents and children most frequently mentioned the mental function domain. Additionally, parents frequently described problems with social skills (interpersonal interactions and relationships [n = 20, 21%]), and children often mentioned learning difficulties (n = 41, 45%). Movement-related difficulties were more often expressed by parents (n = 20, 21%) than children (n = 8, 9%). When analyzing the ICF-coded categories up to the fourth-digit level, movement, learning abilities, and mental functions were the most frequently reported difficulties (Table II, Table III). Eight participants (HIE: n = 1, PAIS: n = 7) and 23 parents (HIE: n = 12, PAIS: n = 11) reported no difficulties.

Table IV demonstrates the difficulties for children with a clinically diagnosed cognitive delay (n = 18) or motor delay/CP (n = 27) vs those without. The categories of mentioned difficulties were quite similar (memory, attention, and movement difficulties) for both participants with and without a cognitive delay, with the percentages of participants reporting difficulties being higher in those with a cognitive delay. The most frequently mentioned difficulties by children with a motor delay were on movement, independence, and general learning, while those without most often mentioned cognitive functions.

**Table IV:** Top-mentioned difficulties in daily functioning sorted by reporting frequency of participants with and without a cognitive or motor delay diagnosed during clinical follow-up, and specified by self-report and parent-proxy report

The reported difficulties varied between HIE and PAIS participants. HIE participants commonly noted issues with attention, processing speed, and impulse control, while PAIS participants highlighted movement, planning, energy levels, and sensory processing. Difficulties with general learning and memory were frequent in both groups. No differences between children and young adults were observed in the PAIS cohort.

### Impact on Daily Life

Approximately half of the parents of HIE (n = 26) and PAIS (n = 24) participants reported that the reported difficulties significantly impacted their lives (Table III). Parents of HIE participants mentioned their children needing additional help and guidance in daily activities, having difficulty keeping up at school, and negatively influencing the family atmosphere. Parents of PAIS participants mainly observed behavior and emotional regulation issues. PAIS participants required more assistance, time, and explanation for tasks and had limited energy. Older PAIS participants primarily reported experiencing anger, irritation, insecurity, and fear; needing help with daily activities; and often feeling their struggles were overlooked.

### Concerns and Barriers for Development

Parents of HIE participants identified their child's attention and behavioral difficulties as the primary barriers for development. Additionally, they were concerned about their child's future independence, and their ability to engage in daily activities. Parents of PAIS participants expressed concerns about their child's independence and about their child's feelings of fear, insecurity, or shame. Participants themselves also highlighted concerns about their independence, their emotions or behavior, and the challenges in attending school and learning.

## Discussion

This cross-sectional questionnaire study explored the daily functioning of children, adolescents, and young adults with a history of PAIS or HIE and TH by self-report and parent-proxy report. As strengths, the participants most often reported their participation in recreational activities and school-related learning abilities, while parents most frequently commented on their child's personality traits. Over 3 quarters of the participants reported difficulties in daily functioning, primarily concerning movement, learning, and cognitive functions.

To the best of our knowledge, this is the first questionnaire study to examine the daily functioning of children after HIE or PAIS by using both self-reports and parent-proxy reports. Previous questionnaire studies regarding outcome of infants with a complicated birth history mainly focused on parent-proxy reports of very preterm infants.^13^^,^^14^ Jansen et al investigated social-emotional and behavioral issues at 2 and 10 years of age after very preterm birth.^13^ The positive aspects reported by parents at age 10 most often concerned the theme personality style, in accordance with the parent-reported strengths in our study. A questionnaire study of Jaworski et al examined parental perspectives on outcome at 18 months’ corrected age following very preterm birth. Parents most frequently mentioned personality (61%), happiness (40%), and developmental outcome/progress (40%) as positive things about their child.^14^

Regarding parental concerns, Jaworski et al most often reported concerns on neurodevelopment, particularly language, behavior, and physical health.^14^ Jansen et al reported that parents' main concerns at 2 years related to developmental milestones, development in relation to the self and others, and physical development. At 10 years of age, however, these concerns shifted, with a stronger focus on development in relation to the self and others, particularly regarding emotional development.^13^ In our study, parents often mentioned neuromusculoskeletal and movement-related difficulties, together with cognitive and learning problems. As children encounter increasingly complex developmental demands across multiple domains while growing older, parents' main concerns might also shift more to the cognitive domain. A child's age might therefore influence the type of answers parents give with regard to functioning.

Our study identified similarities in the strengths and difficulties experienced by HIE and PAIS participants. Difficulties in cognitive functioning were frequently reported in both groups. Although brain injury following HIE may be more strongly associated with cognitive impairments due to its diffuse nature, localized injuries from PAIS could also result in cognitive difficulties by disrupting critical developmental networks. However, we also observed differences. PAIS participants more frequently reported movement-related difficulties and reduced energy levels, which may be attributed to the higher prevalence of CP in that population.^15^ Concerning cognition, PAIS participants reported a pattern of challenges related to information processing and planning and organization, while HIE participants more commonly described difficulties with attention, processing speed, and impulse control. As our study design did not permit causal inferences, future research is needed to determine whether these differences stem from the distinct etiologies of perinatal brain injury.

We also observed differences between self-reports and parent-proxy reports. For instance, parents reported movement difficulties almost as frequently as a movement delay or CP was diagnosed during clinical follow-up visits, while participants rarely mentioned it. In contrast, parents more frequently reported no difficulties at all compared to the participants themselves (23% vs 8%, respectively). Schiariti et al previously reported the strengths and limitations in functioning important to children with CP aged 4-16 years using semistructured interviews and compared self-reports with caregiver-proxy reports by using components of the ICF-CY.^16^ In contrast to our findings, children generally had a positive view of their abilities and primarily described their strengths and facilitating factors, while caregivers focused more on limitations. As children grow older, they may experience more difficulties as they can grow into their deficits, and as their ability for self-reflection develops.^17^ The inclusion of younger children (4-8 years) in that cohort may explain their more positive view on functioning. Differences between parent-proxy and self-reports in our study might be explained by the way the questions were formulated. Parents were asked about difficulties in daily functioning related to perinatal brain injury, which was considered too complex for children. Consequently, children were asked what they found difficult to do in general, which might have yielded a higher rate of self-reported difficulties.

This study on the consequences of perinatal brain injury from the perspective of patients and their caregivers allows us to reflect on parental counseling and neurodevelopmental surveillance. While motor functions are often repeatedly assessed from an early age, certain cognitive skills that were frequently mentioned among the difficulties in this study (eg, memory and attention) are often not specifically assessed. We observed that participants without a diagnosed cognitive or motor delay still frequently reported difficulties related to cognitive functioning. Previous studies using standardized tests and parental questionnaires revealed that as children with a history of HIE and PAIS grow older, they often face a variety of challenges beyond motor difficulties, including cognitive, behavioral, socio-emotional, and audiovisual problems.^2^^,^^5^^,^^18^, ^19^, ^20^ Although neuroimaging with MRI may facilitate the provision of more individualized outcome information to parents,^21^ it is important that clinicians and researchers actively engage with parents and children during follow-up to explore the full spectrum of possible consequences of perinatal brain injury, tailoring the addressed topics to the child's developmental stage and individual needs.

In addition to guiding clinical counseling and surveillance improvements, our results also contribute to the identification of outcomes that are of importance to the stakeholders in medical research. Patients’ perspectives are increasingly incorporated to define outcomes and ensure their relevance, for example, through standard outcome sets that include patient- or proxy-reported outcome measures.^22^^,^^23^ While not intended to establish patient reported outcome measures, this study provides an initial understanding of the perspectives of children with perinatal brain injury.

### Limitations

Our study has several limitations. Of all eligible HIE and PAIS patients, 48% and 34% participated. However, no significant differences in neonatal and MRI characteristics between the participants and nonparticipants were identified, except for gestational age in the PAIS cohort. Furthermore, 32 participants had comorbidities which possibly influenced daily functioning, and neurodevelopmental impairments may have been underdiagnosed, as some children last visited follow-up before reaching school age. Moreover, it was not possible to retrospectively determine whether there were minor motor problems that could explain the challenges in movement that were reported among the participants without a motor delay. The design of this single-center study, with a digital questionnaire filled out voluntarily and requiring understanding of English or Dutch, may have resulted in selection bias. The decision to participate may have been influenced by (limited) access to digital devices, and the severity of impairment of the child. Furthermore, as we did not include a control group, it is uncertain whether the reported strengths and difficulties are different from children and young adults without perinatal brain injury. Therefore, we chose to report on the issues that were mentioned among at least 10% of the study population. Another limitation includes the age difference between the HIE and PAIS participants, as TH was implemented in our hospital in 2008 and HIE participants therefore had a maximum age of 16 years. Although this might have contributed to some differences that were observed between these 2 populations, there were no apparent differences in the reported difficulties between children and young adults within the PAIS group.

Several factors might limit the generalizability of our findings. Although socioeconomic status scores among participants and nonparticipants were similar, they were slightly above average. Additionally, when comparing the characteristics of our cohort with trial cohorts on TH, one could reason that participants seem relatively mildly affected.^24^^,^^25^ Fifteen HIE participants had mild HIE, and only 1 had severe HIE according to Sarnat staging. We, however, believe this cohort is representative of the HIE population in our country. Over the last decade, the use of TH has increased among infants with mild HIE^26^ and severe HIE often results in redirection of care in case of severe brain injury on MRI. Furthermore, the Sarnat stage did not differ between the participants and nonparticipants with HIE.

## Conclusions

Despite the challenges that children, adolescents, and young adults with a history of perinatal brain injury face, they frequently report thriving in recreational activities and learning pursuits, highlighting their abilities and resilience. Parents most often described their children's strengths in terms of personality, social skills, and learning abilities. Simultaneously, the frequently experienced difficulties including problems in movement, learning, and cognitive functions should encourage health care professionals and researchers to pay attention to the full spectrum of the possible consequences of perinatal brain injury, including subtle and hidden impairments, in neurodevelopmental surveillance and parental counseling. These findings emphasize the importance of proper neurodevelopmental surveillance, counseling, and support throughout childhood and adolescence, with acknowledgement of the resilience of the children and their families.

## CRediT authorship contribution statement

**Lisanne M. Baak:** Writing – review & editing, Writing – original draft, Project administration, Methodology, Investigation, Formal analysis, Data curation, Conceptualization. **Corline E.J. Parmentier:** Writing – review & editing, Writing – original draft, Project administration, Methodology, Investigation, Formal analysis, Data curation, Conceptualization. **Nienke Wagenaar:** Writing – review & editing. **Fleur-Sophie N. Buijsen:** Writing – review & editing, Project administration. **Anne A.M. van Wendel de Joode:** Writing – review & editing. **Jan Willem Gorter:** Writing – review & editing, Supervision, Methodology, Conceptualization. **Manon J.N.L. Benders:** Writing – review & editing, Supervision, Methodology, Conceptualization. **Floris Groenendaal:** Writing – review & editing, Supervision, Methodology, Conceptualization. **Linda S. de Vries:** Writing – review & editing, Supervision, Methodology, Conceptualization. **Cora H. Nijboer:** Writing – review & editing. **Thomas Alderliesten:** Writing – review & editing. **Corine Koopman-Esseboom:** Writing – review & editing. **Cornelia H. Verhage:** Writing – review & editing. **Rian M.J.C. Eijsermans:** Writing – review & editing. **Niek E. van der Aa:** Writing – review & editing, Supervision, Methodology, Investigation, Funding acquisition, Formal analysis, Conceptualization. **Marjolijn Ketelaar:** Writing – review & editing, Supervision, Methodology, Investigation, Formal analysis, Conceptualization.

## Data Statement

Data sharing statement available at www.jpeds.com.

## Declaration of Competing Interest

L.M.B. reports financial support was provided by the Wilhelmina Children's Hospital, University Hospital Center Utrecht, which had no role in the study design; the collection, analysis, and interpretation of data; the writing of the report; or the decision to submit the paper for publication. The other authors declare that they have no known competing financial interests or personal relationships that could have appeared to influence the work reported in this paper.
