# Supplementary material for: Child and Parent Perspectives on Daily Functioning after Perinatal Brain Injury
Source: J Pediatr Clin Pract. 2025 Aug 7;17:200172. doi: 10.1016/j.jpedcp.2025.200172 (PMC12398854; doi:10.1016/j.jpedcp.2025.200172)
Supplement: Appendix 1 [file mmc1.docx]

## Appendix 1 – Supplementary Methods

Neurodevelopmental surveillance for both the PAIS and HIE cohort consisted of assessment of motor performance, cognition, and behavior using standardized tests. Moreover, the presence of language developmental delay, sensory deficits, and epilepsy were explored.

Motor outcome at 2 years of age was assessed using the motor composite score of the Dutch version of the Bayley scales for Infant and Toddler Development, Third Edition (Bayley-III-NL), or the Griffiths Scales of Child Development for patients in the NSRU born before 2009. At 5-6 years of age, the Dutch version of the Movement Assessment Battery for Children–Second edition (MABC-2-NL) was used. At 8-10 years of age, the second edition of the Bruininks-Oseretsky Test of Motor Proficiency or the MABC-2-NL was used. The severity of CP was classified according to the Gross Motor Function Classification System (GMFCS). Cognitive outcome at 2 years was assessed using the cognitive composite score of the Dutch version of the Bayley-III-NL. At 5-6 years the Wechsler Preschool and Primary Scale of Intelligence III or IV was used. At 8-10 years, the Wechsler Intelligence Scale for Children, Third edition (WISC-III-NL) or Fifth edition (WISC-V-NL) were used. Behavioral problems were identified with the Child Behavior Checklist, a standardized parental questionnaire reporting on emotional, social, and behavioral problems.

A delay in motor development was defined as a Bayley-III-NL motor composite score or Griffiths developmental quotient <-1 SD, MABC-2-NL total percentile ≤5th percentile, or BOT-2 total motor composite score <-1 SD. A cognitive delay was defined as a cognitive score <-1 SD (Bayley-III-NL cognitive composite score <85, WPPSI-III-NL and WISC-III-NL total IQ, verbal IQ, performance IQ, and/or processing speed <85, and WISC-V-NL full scale IQ, verbal comprehension, visual spatial index, fluid reasoning index, working memory index and/or processing speed index <85). An adverse behavioral outcome was defined as T-scores ≥64 on the total, internalizing, and/or externalizing scale of the Child Behavior Checklist (CBCL) filled out by parents and/or teachers or when serious concerns on behavior was expressed by the healthcare physician. A language developmental delay was defined as a delay according to a Dutch language developmental questionnaire filled out by parent(s), or by the judgement of the neonatologist. Auditory or visual deficits related to perinatal brain injury were defined as a deficit diagnosed by an ophthalmologist or audiologist with requirement for additional support (e.g. hearing aids). Epilepsy diagnosis was defined according to the International League Against Epilepsy definition, with EEG being performed in case of a clinical suspicion of epilepsy.
