# Supplementary material for: Child and Parent Perspectives on Daily Functioning after Perinatal Brain Injury
Source: J Pediatr Clin Pract. 2025 Aug 7;17:200172. doi: 10.1016/j.jpedcp.2025.200172 (PMC12398854; doi:10.1016/j.jpedcp.2025.200172)
Supplement: Appendix 2 [file mmc2.docx]

## Appendix 2 – Supplementary Tables

**Supplementary Table 1: Overview of the online questionnaire for the parents, and the children and young adults with a history of perinatal brain injury.**

|  | ***Parents*** | ***Children*** | ***Young adults*** |
| --- | --- | --- | --- |
| *Q1* | *Can you tell us about the strengths of your child?* | *Can you tell us something about the things you are good at?* | *Can you tell us something about your strengths or the things you are good at?* |
| *Q2* | *Are there things in daily life your child has difficulties with, of which you think they might relate to the brain injury around birth?* | *What things do you find difficult or are hard for you?* | *Are there things in your daily life you find difficult, of which you think they might relate to the brain injury around birth?* |
| *Q3* | *Do the things you indicated at Q2 influence the daily life of your child or your family? If so, in what way?* | *-* | *Do the things you indicated at Q2 influence the daily life of you or your family? If so, in what way?* |
| *Q4* | *What is the most important barrier or concern for the development of your child?* | *Do you worry about now or the future? If so, what do your worry about?* | *What is the most important barrier in your daily life, or what do you worry about?* |

**Supplementary Table 2: Strengths and difficulties depicted in the main ICF-CY categories (one-level digit coding) and sorted by reporting frequency by the participants and their parents.**

| **PAIS (n=52)** | | | | | | **HIE (n=50)** | | | | | | **Total (n=102)** | | | | | |
| --- | --- | --- | --- | --- | --- | --- | --- | --- | --- | --- | --- | --- | --- | --- | --- | --- | --- |
| **Strengths** | | | | | | | | | | | | | | | | | |
| **Parent (n = 44)** | | | **Child (n = 46)** | | | **Parent (n = 50)** | | | **Child (n = 45)** | | | **Parent (n = 94)** | | | **Child (n = 91)** | | |
| Mental functions | 42 | 95% | Community, social and civic life | 31 | 67% | Mental functions | 39 | 78% | Community, social and civic life | 36 | 80% | Mental functions | 81 | 86% | Community, social and civic life | 67 | 74% |
| Interpersonal interactions and relationships | 19 | 43% | Learning and applying knowledge | 20 | 43% | Interpersonal interactions and relationships | 27 | 54% | Learning and applying knowledge | 27 | 60% | Interpersonal interactions and relationships | 46 | 49% | Learning and applying knowledge | 47 | 52% |
| Community, social and civic life | 18 | 41% | Mental functions | 12 | 26% | Learning and applying knowledge | 22 | 44% | Interpersonal interactions and relationships | 8 | 18% | Learning and applying knowledge | 38 | 40% | Interpersonal interactions and relationships | 19 | 21% |
| Learning and applying knowledge | 16 | 36% | Interpersonal interactions and relationships | 11 | 24% | Community, social and civic life | 18 | 36% | Mental functions | 7 | 16% | Community, social and civic life | 36 | 38% | Mental functions | 19 | 21% |
| Domestic life | 12 | 27% | Domestic life | 10 | 22% | Domestic life | 10 | 20% | Domestic life | 6 | 13% | Domestic life | 22 | 23% | Domestic life | 16 | 18% |
| Humor | 7 | 16% | Communication | 3 | 7% | Empathy | 9 | 18% | Communication | 2 | 4% | Humor | 15 | 16% | Communication | 5 | 5% |
| Empathy | 6 | 14% | Humor | 3 | 7% | Humor | 8 | 16% | Humor | 2 | 4% | Empathy | 15 | 16% | Humor | 5 | 5% |
| Communication | 4 | 9% | Neuromusculoskeletal and movement-related functions | 1 | 2% | Communication | 7 | 14% | Neuromusculoskeletal and movement-related functions | 2 | 4% | Communication | 11 | 12% | Neuromusculoskeletal and movement-related functions | 3 | 3% |
| Neuromusculoskeletal and movement-related functions | 1 | 2% | Major life areas | 1 | 2% | Neuromusculoskeletal and movement-related functions | 4 | 8% | Major life areas | 2 | 4% | Neuromusculoskeletal and movement-related functions | 5 | 5% | Major life areas | 3 | 3% |
| General tasks and demands | 1 | 2% | Empathy | 1 | 2% | General tasks and demands | 1 | 2% | Self-care | 2 | 4% |  |  |  | Self-care | 2 | 2% |
| **Difficulties** | | | | | | | | | | | | | | | | | |
| **Parent (n = 44)** | | | **Child (n = 46)** | | | **Parent (n = 50)** | | | **Child (n = 45)** | | | **Parent (n = 94)** | | | **Child (n = 91)** | | |
| Mental functions | 24 | 55% | Mental functions | 20 | 43% | Mental functions | 31 | 62% | Learning and applying knowledge | 28 | 62% | Mental functions | 55 | 59% | Mental functions | 44 | 48% |
| Neuromusculoskeletal and movement-related functions | 12 | 27% | Learning and applying knowledge | 13 | 28% | Interpersonal interactions and relationships | 11 | 22% | Mental functions | 24 | 53% | Interpersonal interactions and relationships | 20 | 21% | Learning and applying knowledge | 41 | 45% |
| Interpersonal interactions and relationships | 9 | 20% | Neuromusculoskeletal and movement-related functions | 8 | 17% | Learning and applying knowledge | 10 | 20% | Interpersonal interactions and relationships | 7 | 16% | Neuromusculoskeletal and movement-related functions | 20 | 21% | Interpersonal interactions and relationships | 13 | 14% |
| Learning and applying knowledge | 9 | 20% | Interpersonal interactions and relationships | 6 | 13% | Neuromusculoskeletal and movement-related functions | 8 | 16% | Community, social and civic life | 7 | 16% | Learning and applying knowledge | 19 | 20% | Community, social and civic life | 11 | 12% |
| Sensory functions and pain | 8 | 18% | Sensory processing | 5 | 11% | Sensory functions and pain | 8 | 16% | Communication | 3 | 7% | Sensory functions and pain | 16 | 17% | Neuromusculoskeletal and movement-related functions | 8 | 9% |
| Sensory processing | 7 | 16% | Community, social and civic life | 4 | 9% | Communication | 4 | 8% | Self-care | 2 | 4% | Sensory processing | 10 | 11% | Communication | 6 | 7% |
| Independence | 4 | 9% | Communication | 3 | 7% | Sensory processing | 3 | 6% | General tasks and demands | 2 | 4% | Independence | 7 | 7% | Sensory processing | 5 | 5% |
| General tasks and demands | 3 | 7% | Major life areas | 3 | 7% | Independence | 3 | 6% | Major life areas | 1 | 2% | General tasks and demands | 6 | 6% | Self-care | 4 | 4% |
| Community, social and civic life | 2 | 5% | Independence | 3 | 7% | General tasks and demands | 3 | 6% | Sensory functions and pain | 1 | 2% | Communication | 5 | 5% | General tasks and demands | 4 | 4% |
|  |  |  | Self-care | 2 | 4% | Self-care | 2 | 4% | Mobility | 1 | 2% | Self-care | 3 | 3% | Major life areas | 4 | 4% |
|  |  |  | Sensory functions and pain | 2 | 4% |  |  |  |  |  |  |  |  |  | Sensory functions and pain | 3 | 3% |
|  |  |  | General tasks and demands | 2 | 4% |  |  |  |  |  |  |  |  |  | Independence | 3 | 3% |

PAIS: perinatal arterial ischemic stroke, HIE: hypoxic-ischemic encephalopathy
